# Editorial: Diagnostic, prognostic and treatment efficacy power of biomarkers of aging for frailty, age-related diseases and multimorbidity

**DOI:** 10.3389/fendo.2025.1574133

**Published:** 2025-03-10

**Authors:** Stefano Salvioli, Florencia M. Barbé-Tuana, Maria Conte

**Affiliations:** ^1^ Department of Medical and Surgical Sciences (DIMEC), University of Bologna, Bologna, Italy; ^2^ IRCCS Azienda Ospedaliero-Universitaria di Bologna, Bologna, Italy; ^3^ School of Sciences, Health and Life at Pontifical Catholic University Rio Grande do Sul, Porto Alegre, Brazil

**Keywords:** aging, biomarkers, age-related diseases, frailty, geroscience

Geroscience, a new branch of geriatrics and gerontology, postulates that organismal aging and age-associated diseases share the same basic molecular mechanisms. According to this assumption, there is no actual boundary between the aging process and a number of non-communicable diseases (NCDs) associated with age (1, 2). Consequently, aging turns to be the major modifiable driver of age-related NCDs, as well as other late-life conditions, including frailty (3).

Based on the above concepts, it becomes clear that the identification and validation of biomarkers of age able to classify people as biologically older (or younger) than their chronological age is becoming of paramount importance (4). In fact, subjects characterized by a biological age higher than the chronological one are at higher risk of developing of many age-associated diseases, including cardiovascular diseases, neurodegeneration, cancer, etc. In turn, patients suffering from these diseases are biologically older than healthy age-matched individuals.

**Figure 1:**
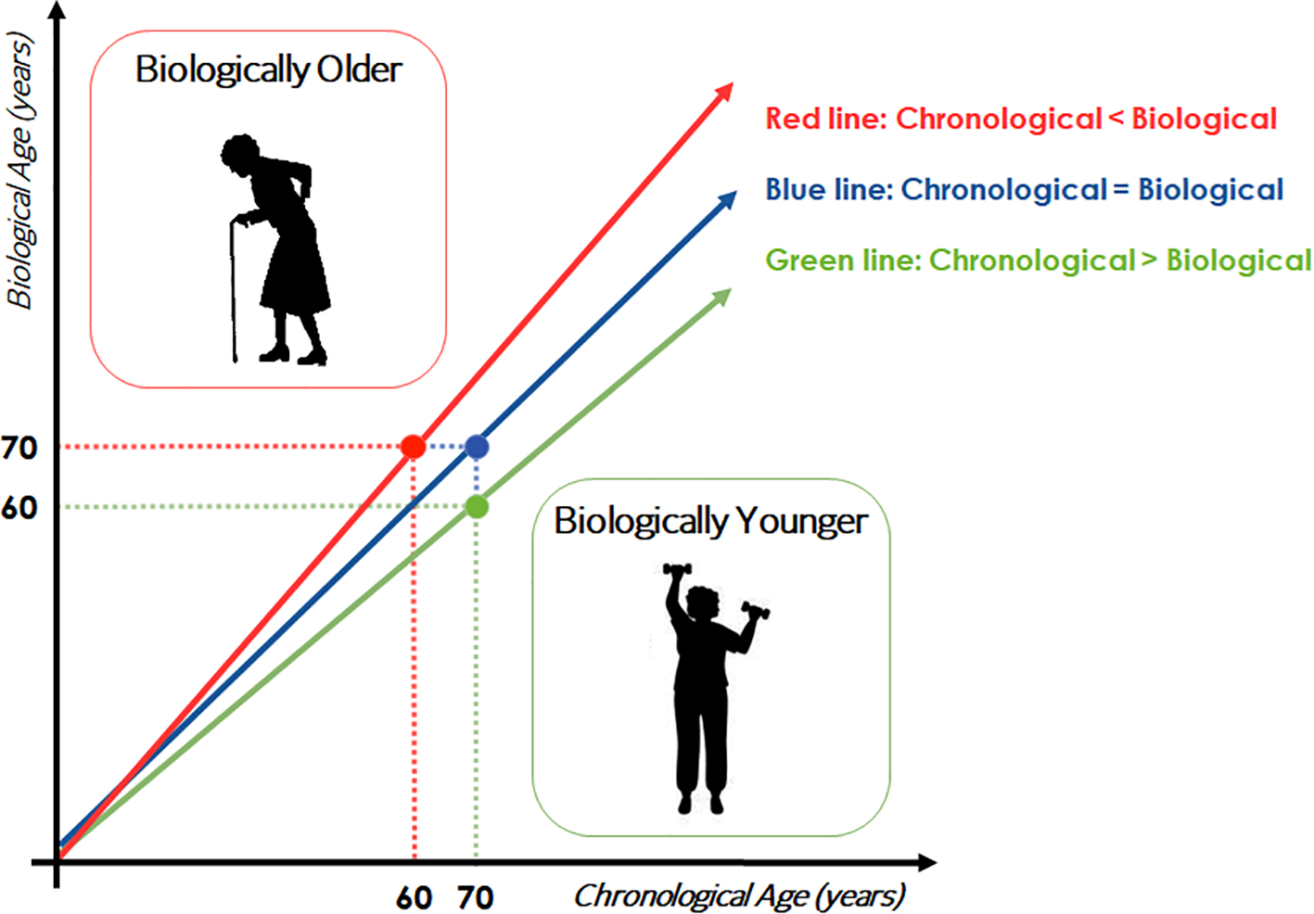
Schematic representation of the concept of biological age. Trajectories of a hypothetical biomarker of age are showed.

Biological age can be assumed as the age of an individual defined by the level of age-dependent biological changes, such as accumulation of molecular and cellular damage (5). Biological age may differ from chronological (= calendar) one. In particular, biological age can be summarized as the age matching the chronological one at which the average reference population shares the individual’s level of the age-dependent biological parameter considered (**Figure 1**). In this case, an example is given with a hypothetical parameter that constantly increases with age. Of course, the opposite situation can also occur. In this case, a 70-year-old subject represented in the figure with the green circle has a level of the considered parameter similar to that found in a 60-years-old population, so he/she can be considered biologically younger than subjects of his/her birth cohort. The opposite can be said for the subject indicated with the red circle, who is therefore biologically older. A more complicated situation can happen when considering parameters that do not change monotonically, but instead have fluctuations across age groups. As an example, some parameters may display peaks at 30, 60 and 80 years of age (6). In this case, such parameters are likely not useful as indicators of biological age. Hypothesizing the existence of a threshold value of biological age from which frailty and morbidities occur, a straight-forward consequence is that biologically younger individuals will become frail much later in life, and will therefore stay healthy longer. The validity of these considerations for a single parameter/biomarker has to be taken with caution, since different biomarkers can provide different or even opposite results for the same individual. It is therefore advised to consider more parameters simultaneously to be sure about the indication on the biological age (and consequent acceleration or deceleration over chronological age). This is a rapidly expanding field of research, with evident consequences and interests not only in medicine and geriatrics, but also in health care policy and business.

With this Research Topic dedicated to the investigation on the diagnostic, prognostic and treatment efficacy power of biomarkers of aging for frailty, age-related diseases and multimorbidity, published in Frontiers of Endocrinology between 2023 and 2024, we aimed to offer a stage to forefront studies in this field. Although it was certainly out of the scope of this Research Topic to cover all the studies that are performed worldwide on this matter, it is however able to provide the reader with a glimpse of the vastity and variety of fields in which biomarkers can be applied in age-associated conditions. It is however to note that not in all cases the biomarkers reported in these papers have been previously demonstrated to be formally associated with age, so it is possible that some of them maintain the association with the observed outcome only in the particular condition that has been reported in the paper.

Fourteen original research papers are included in this Research Topic, describing results from studies dealing with different aspects of this biomarkers. In particular, soluble biomarkers, including metabolites have been investigated in diverse clinical or real-world settings. Genetics and epigenetics, as well as functional parameters have been investigated, too.

In particular, as far as soluble biomarkers, with a machine learning-based approach Capri et al., identified the most significant blood biomarkers able to discriminate symptomatic from asymptomatic older patients with carotid stenosis (albumin, C-reactive protein, percentage of monocytes, and CXCL9). Valdes et al., identified fibroblast growth factor 23 (FGF-23) and interleukin-15 receptor alpha (IL15RA) as factors associated with both acute kidney injury and post-operative mortality after hip fracture surgery in frail patients. Carbone et al., have investigated the possibility that serum leptin and adiponectin may have a predictive power for the onset of brain infarcts in a population of patients with either Mild Cognitive Impairment or Alzheimer’s Disease. On the same vein, Ingannato et al., reported that GFAP, NfL and pTau 181 levels in plasma were lower in patients with Subjective cognitive decline and Mild cognitive impairment than in AD patients. Chiariello et al., reported on the circulating as well as intramuscular levels of GDF15 in sarcopenic patients and, while the intramuscular form seemed associated with muscle health, the circulating form was associated with decreased Isometric Quadriceps Strength and IGF-1 levels. Liu et al., identified an association between adiponectin, leptin, and retinol-binding protein-4 (RBP-4) levels and the risk of metabolic dysfunction-associated fatty liver disease, which in turn is associated with testosterone deficiency in old Taiwanese men. Moving from proteins to metabolites, Wu et al., performed plasma metabolomics through untargeted LC/GC-MS in a population of osteoporotic older patients and identified 33 differential metabolites in elderly men and 30 differential metabolites in elderly women that could be potential biomarkers for osteoporosis, while Long et al., identified three blood metabolites serving as causal mediators in the context of delirium of hospitalized older patients (LDL-C, sphingomyelin, and O-methylascorbate).

Finally, Mao et al., found that preoperative serum levels of oxaloacetate and 2-aminoadipic acid were associated with postoperative delayed neurocognitive recovery following general anesthesia in older patients.

As far as genetics and epigenetics, Damanti et al., identified some SNPs related to frailty and linked to the renin–angiotensin system, apoptosis pathways, growth hormone signaling, inflammation, adducin, and the 9p21–23 region to be associated with various measures of obesity in community-dwelling older adults, while Gialluisi et al. reported a small but significant increase of ΔPhenoAge in prevalent Parkinson’s Disease cases vs healthy subjects. Last but not least, two papers described results obtained on functional parameters: Montesanto et al., reported that Short Physical Performance Battery (SPPB) is the most important risk factor for mortality following a 5-year follow-up in Type 2 Diabetic patients among age, sex, SPPB, chronic kidney disease, myocardial ischemia, peripheral artery disease, neuropathy, and myocardial infarction, while Choi et al., associated the metabolic activity of inflamed visceral adipose tissue with severity of age-related macular degeneration through 18F-fluorodeoxyglucose (FDG) positron emission tomography/computed tomography. Finally, Xu et al., introduced the concept of dynamic FT3 changes to forecast mortality in older patients in Intensive Care Units.

As a whole, this Research Topic helps to shed light on the emerging topic of biomarkers of age as useful tools to identify people and patients at risk for specific conditions or, more in general, for frailty and mortality, and to evaluate the efficacy of preventive or therapeutic treatments, not to mention the possibility that these biomarkers are *per se* possible targets for anti-aging treatments. More extended and (possibly) longitudinal studies will be needed to confirm whether the findings reported in these papers could be translated into clinical practice for diagnostic and prognostic purposes.
